# A Rare Case of Biopsy-Proven Sacral Mass Metastatic From Follicular Thyroid Cancer With a Literature Review

**DOI:** 10.7759/cureus.64730

**Published:** 2024-07-17

**Authors:** Aniqa Faraz, Kehua Zhou, Faraz A Tariq

**Affiliations:** 1 Internal Medicine, Cumberland Medical Center, Crossville, USA; 2 Internal Medicine, ThedaCare Regional Medical Center, Appleton, USA; 3 Dentistry, University of Tennessee Medical Center, Knoxville, USA

**Keywords:** soft tissue metastasis, cancer metastasis, follicular thyroid cancer, thyroid cancer, sacral mass

## Abstract

Thyroid cancer with metastatic disease to the pelvis is extremely rare. The patient in our case, an 86-year-old male, presented after total thyroidectomy for follicular thyroid cancer (FTC) with symptoms of recurrent urinary tract infections and retentions, surprisingly leading to the discovery of a large sacral mass on the CT abdomen and pelvis. The biopsy showed metastatic carcinoma with morphology and immunohistochemistry to be consistent with FTC. In our case, due to symptomatology, prostate cancer was initially considered high in the differential for primary source rather than thyroid cancer. The mass was considered too large for surgery, and he was referred to a radiation oncologist for radiation therapy for the sacral mass.

## Introduction

Follicular thyroid cancer (FTC) is the second most common subtype of thyroid cancer after papillary thyroid cancer (PTC) and accounts for about 10% of all thyroid cancers. FTC is a thyroid follicular epithelial cell neoplasm with evidence of capsular and/or vascular invasion. The characteristic features of PTC are papillary architecture, the presence of psammoma bodies, and/or characteristic nuclear features (nuclear chromatin, nuclear orientation, and grooving). The incidence of distant metastasis in FTC is 6-20% and is common to the lungs and bones. Distant metastasis in FTC is poorly studied and is associated with significant morbidity and mortality [1]. Soft tissue metastasis is extremely rare. FTC is predominant in females and spreads hematogenously, which explains the more distant metastasis with this subtype as compared to PTC [1-3]. Distant metastasis is an independent risk factor and the most important prognostic factor for FTC mortality. Wu et al. analyzed a prospectively maintained dataset of 190 patients with FTC and concluded that FTC patients without metastatic disease had a cancer-specific survival rate of 100%. On the other hand, the outcomes of patients with metastatic disease at presentation or during follow-up were 26.0% and 76.6%, respectively [4].

We present a sporadic case of an 86-year-old male with a history of FTC status post thyroidectomy and iodine 131 treatment who was referred to a urologist for urinary symptoms and found to have a sacral mass on the computed tomography (CT) abdomen and pelvis. This case emphasizes the importance of early metastatic workup for pelvic mass detected incidentally, which can be extremely beneficial in early diagnosis and treatment. Oncologists should be diligent in closer bone and soft tissue mass surveillance for all follicular thyroid cancer patients. 

## Case presentation

An 86-year-old white male with a past medical history of diabetes mellitus, hypertension, hypothyroidism, thyroid cancer, prostate cancer, and GERD presented to the urologist referred by a primary care physician for frequent symptoms of urinary difficulty and recurrent urinary tract infections (UTIs) and was found to have a sacral mass on CT abdomen and pelvis. He denied a change in appetite, weight loss, or back pain at that time though later after a few weeks he started developing sacral pain. Medical history revealed thyroidectomy and iodine 131 treatment for thyroid cancer about 20 years ago, and prostate cancer status post radiation therapy with a disease-free duration of about 10 years. 

He had been a former pipe smoker and denied any alcohol use. His family history was positive for pancreatic cancer in his father. Physical examination did not reveal any abnormalities other than a lumbar scar from previous back surgery for disc disease. No swelling or tenderness was appreciated on the back. The basic laboratory workup in this case is listed in Table 1.

**Table 1:** The laboratory findings of this case. BUN: Blood urea nitrogen; TSH: thyroid-stimulating hormone

Tests	Results	Reference Range
White blood count	4100 cells/microliter(mcL)	4000-11000 cells/mcL
Hemoglobin	14.4 grams/deciliter(g/dL)	13.8 to 17.2 g/dL
Hematocrit	44.4%	40-54%
Mean corpuscular volume (MCV)	91.2 femtoliters (fl)	80-100 fL
Platelets	88,000 cells/mcL	150,000-450,000 cells/mcL
Neutrophil	88.4%	40-60%
Lymphocyte	11.9%	20-40%
Sodium	136 milliequivalents/liter	135-145 mEq/L
Potassium	4.7 mEq/L	3.5-5.2 mEq/L
Chloride	99 millimoles/liter	96-106 mmol/L
Glucose	337 milligram/deciliter (mg/dL)	70-100 mg/dL
BUN	15 mg/dL	6-24 mg/dL
Creatinine	0.98 mg/dL	0.7-1.3 mg/dL
Calcium	9 mg/dL	8.5-10.2mg/dL
Bilirubin	0.5 mg/dL	0-0.3 mg/dL
Alanine aminotransferase	25 International units/liter (IU/L)	10-50 IU/L
Aspartate aminotransferase	68 IU/L	0-35 IU/L
Alkaline phosphatase	175 IU/L	20-140 IU/L
TSH	3.6 milliunits/L (mU/L)	0.4 and 4.5 mU/L
Prostate specific antigen (PSA)	0.08 nanogram/ml	1 to 1.5 ng/ml

The initial suspicion was for recurrent prostate cancer and cystoscopy was planned, but due to minimal elevation of PSA, a positron emission tomography computed tomography (PET-CT) scan was scheduled.  

**Figure 1:**
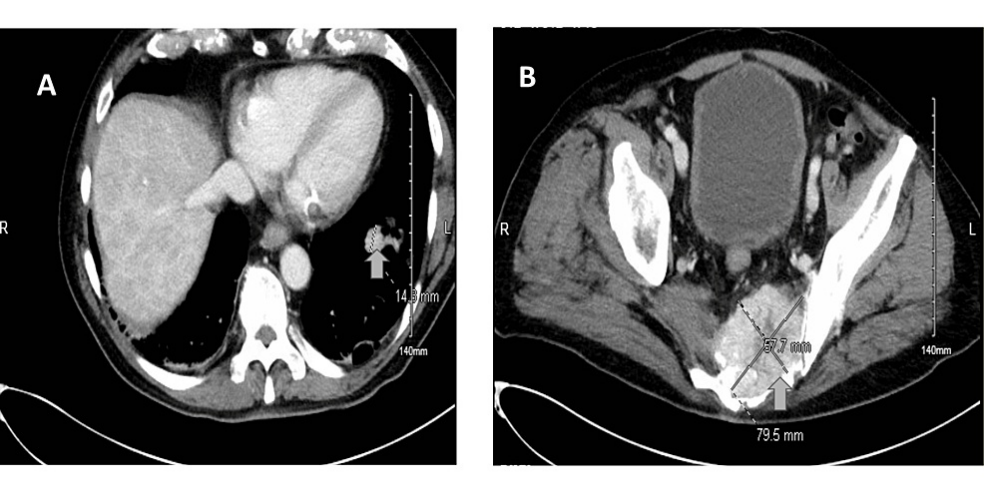
CT abdomen and pelvis with and without contrast revealed (A) pulmonary nodule and (B) 7.9 cm enhancing destructive pelvic mass involving the bilateral sacrum extending into the left pelvic sidewall.

**Figure 2:**
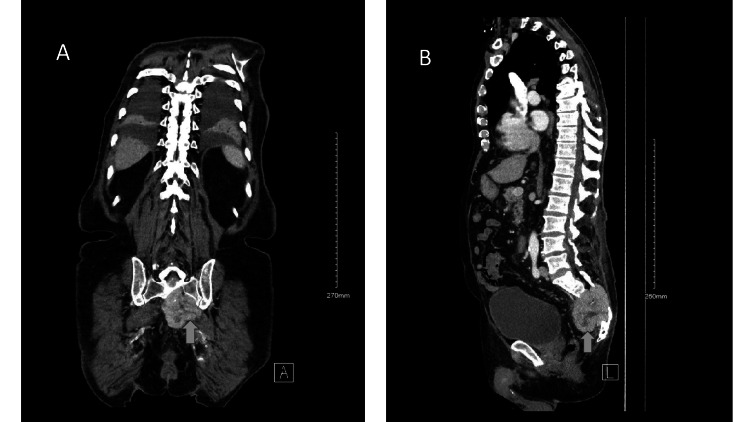
CT abdomen and pelvis with (A) coronal and (b) sagittal images reveal a lobulated sacral mass seen at the sacral vertebrae.

CT abdomen and pelvis with and without contrast revealed 7.9 cm enhancing destructive pelvic mass involving bilateral sacrum extending into the left pelvic sidewall which may represent metastatic disease versus soft tissue sarcoma or other pulmonary neoplasm. Several pulmonary nodules are also seen concerning for metastatic disease (Figures 1, 2). 

**Figure 3:**
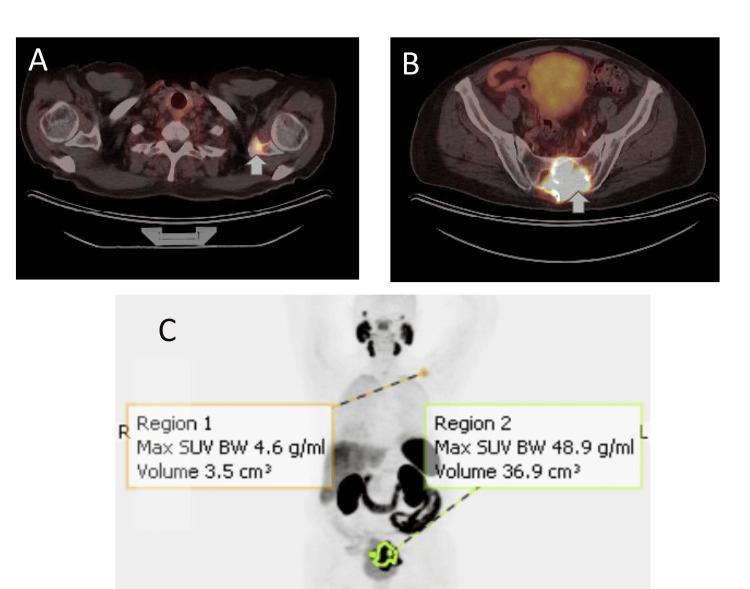
PET-CT scan axial images reveal (A) hypermetabolic sacral mass, (B) hypermetabolic left scapular lesion, and (C) whole body images redemonstrating hypermetabolic regions in the left scapula and sacral region.

The PET-CT scan showed a soft tissue mass lesion associated with the sacrum markedly hypermetabolic with SUV 48.9 consistent with metastasis and there was also a single focus of metastatic disease involving the left scapula. There was no evidence of metastasis elsewhere (Figure 3). A confirmatory biopsy was recommended, and the patient was started meantime on Lupron and Xgeva. 

**Figure 4:**
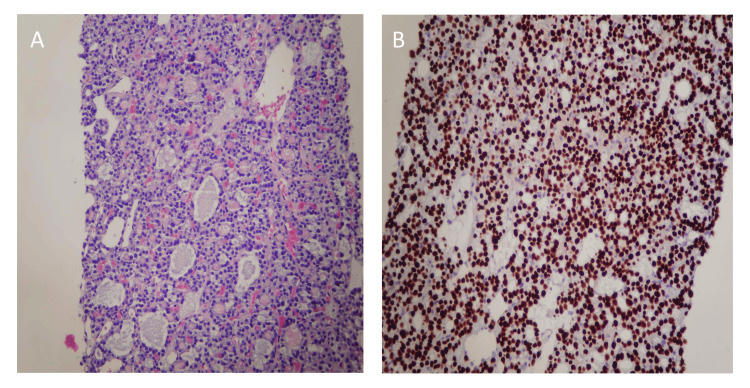
(A) Hematoxylin and eosin (H and E) staining and immunohistochemical staining findings of core biopsy from the sacral mass. (A) H and E staining shows follicular structures with a colloid material. (B) Immunohistochemical (IHC) staining positive with diffuse nuclear expression of thyroid transcription factor 1 (TTF-1).

**Figure 5:**
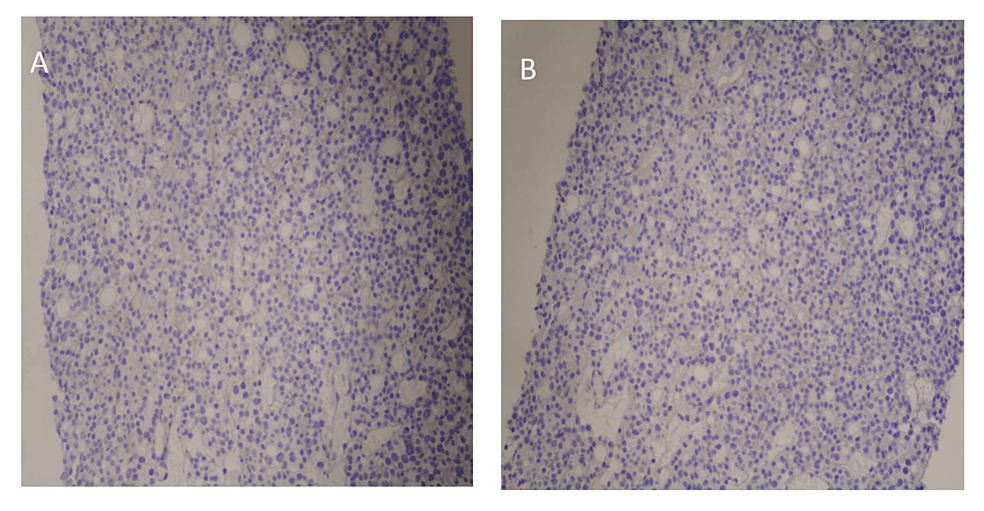
Immunohistochemical staining for PSA and NK3 Homeobox 1 is negative

CT-guided left-sided sacral biopsy was done and pathology showed soft tissue sacral mass biopsy consistent with metastatic thyroid carcinoma follicular variant, associated vascular telangiectasia, no evidence of angiolymphatic invasion or necrosis, and no included bone. Hematoxylin and eosin staining showed core biopsy from the mass exhibiting thyroid follicles and immunohistochemistry (IHC) revealed diffuse nuclear expression of thyroid transcription factor 1 (TTF-1), and IHC for prostate specific antigen (PSA) and NK3 Homeobox 1 was negative as shown in Figures 4, 5.  

The patient has been started on radiation treatment to the sacral area for two weeks and then a month off for recovery followed by two more weeks of localized radiotherapy.

## Discussion

According to the WHO classification, differentiated thyroid carcinomas (DTCs) are divided into papillary and follicular thyroid cancer. Both PTC and FTC derive from thyroid follicular cells. In the United States, about 3 out of 100,000 people will develop a thyroid malignancy and PTC contributes to 80% of all thyroid malignancies [2]. FTC is more common in elderly females and usually presents in 85% of cases as a solitary thyroid nodule and is rarely seen in patients with endemic goiter [3].  

Distant metastasis on initial presentation is detected in 1-3% of cases and is common not only to lungs and bones but also to the liver, kidney, brain, breast, muscle, skin, and at unusual sites such as suprarenal and pelvic soft tissues (as in our case). Distant metastasis is seen in 7-23% of FTC patients and is the most important prognostic factor in the survival of these patients. When seen on initial presentation, it is associated with poor prognosis (10-year survival rate of 50%) [1,4]. 

FTC metastasizes in a hematogenous route by extension into blood vessels, which explains the nature of distant metastasis. Factors involved in fostering metastasis include epithelial mesenchymal plasticity, cancer stem cells, cytokines, noncoding RNAs, and receptor tyrosine kinase pathways [5]. Soft tissue and muscle metastasis from FTC are rare and there is no presenting complaint other than symptoms elicited by the pressure of a growing mass on surrounding tissues [1]. Table 2 summarizes the different metastatic sites that have been reported from FTC at different ages and genders in different case reports.  

**Table 2:** Cases of follicular thyroid cancer metastatic to the soft tissue and muscles indicating different metastatic sites, age, and gender of presentation.

Authors	Patient’s Age /Sex (Male M, Female F)	Metastatic Site	Year
Sevinc et al. [6]	58/F	Scapular Region	2000
Olejarski et al. [7]	73/F	Thigh	2014
Rodrigues and Ghosh [3]	42/M	Scalp, Forearm	2003
Tronnier et al. [8]	53/F	Neck	2009
Tronnier, et al. [8]	66/F	Scalp	2009
Grivas et al. [9]	73/F	Urinary Bladder	2012
Wang et al. [10]	78/F	Buccal Mucosa	2020
Aslan et al. [11]	57/F	Breast	2014
García-Burillo et al. [12]	69/F	Round ligament of liver	2021
Kojima et al. [13]	77/M	Scalp	2023
Ferdous et al. [14]	43/F	Mediastinal	2021
Kumar et al. [15]	45/F	Scalp	2016
Nawarathna et al. [16]	68/M	Chest wall	2016
Piplani et al. [1]	75/F	Pelvic mass	2014
Nayak et al. [17]	67/F	Chest wall	2013
Tong et al. [18]	66/F	Sinonasal tract	2021
Tiong et al. [19]	66/M	Upper thigh	2001

Thyroid nodules are the most common symptom of DTC. Fine needle aspiration cytology (FNAC) is performed if thyroid nodules are suspicious of malignancy. FNAC is useful to identify cytologic features of PTC. However, it is not helpful in making a diagnosis of FTC. The need for further imaging depends on suspicion of the extent of the disease. This may involve neck ultrasound for enlarged cervical lymph nodes, plain chest X-ray for macro-metastasis to lungs and bones, CT or MRI neck for infiltration of trachea or esophagus, and in recurrent disease, other imaging modalities that can contribute to diagnosis are Technetium-99m, Thallium-201, and fluorodeoxyglucose-positron emission tomography [2]. 

Age>45 years is a negative prognostic factor for distant metastasis and its incidence increases with age above 45. The staging for FTC is based on age. Category 1 is below 45 years of age and Category 2 is for above 45 years of age. For those below 45 years of age, if carcinoma is confined to the thyroid only, with no lymph node or distant metastasis, it is stage 1; otherwise, it will be stage 2. This method of staging is the tumor, node, and metastasis method (TNM) and is the official method of staging by the American Joint Commission on Cancer [3,20]. 

The standard treatment for FTC is total thyroidectomy with adjuvant radioiodine therapy. Current guidelines for the treatment of distant metastasis in FTC include surgery, radiotherapy, and Iodine 131 therapy [1,20]. In metastatic DTC, radioactive iodine therapy (RIT) plays a crucial role and prognosis depends on the extent of radioactive iodine uptake by the tumor. However, as in our case, RIT is ineffective for larger metastasis and will be utilized once the tumor size is small. TSH suppression therapy also plays a role in delaying tumor progression, resulting in improved survival [5]. 

Our case involved a patient with metastatic disease to the sacral region about 20 years after treatment of a primary tumor, which is exceedingly rare. To our knowledge, there are only four cases reported so far in the literature with FTC metastatic disease to the soft tissue in the pelvis, as in Piplani et al. with metastatic sacral mass and Tiong et al. with upper thigh mass extending to the pelvic region [1,19]. The interesting feature of our case is the initial presentation of our case with urinary symptoms due to pressure symptoms on the surrounding regions from this large sacral mass. The use of IHC was beneficial in diagnosing the primary source of this tumor mass and should be utilized in all similar cases. 

## Conclusions

In conclusion, increasing importance should be given to distant metastasis in DTC patients. Our case highlights the need to be informed of unusual presentations at unexpected sites by the FTC. As in our case, the patient presented initially with urinary symptoms and was then found to have a sacral mass. It was initially thought to be a metastatic disease from prostate cancer, as it is less commonly reported from thyroid cancer. Our case was worth publishing as it is an extremely rare event, and this case report also highlights that metastatic FTC should be kept in mind as a differential diagnosis of soft tissue masses. We also emphasized the importance of recognizing the hematogenous spread of thyroid cancer to these rare sites. Recognizing this rare metastasis at an earlier stage and subsequently initiating treatment in time can definitely prove beneficial for the patient's survival, as metastasis is associated with a poor prognosis for FTC patients. More awareness is required by physicians, endocrinologists, and oncologists regarding this rare metastasis, as it has a direct bearing on the early diagnosis, the imaging modalities selected, and the management of the patients.
